# Elevation of gangliosides in four brain regions from Parkinson’s disease patients with a *GBA* mutation

**DOI:** 10.1038/s41531-022-00363-2

**Published:** 2022-08-06

**Authors:** Shani Blumenreich, Tamar Nehushtan, Or B. Barav, Jennifer T. Saville, Tamir Dingjan, John Hardy, Maria Fuller, Anthony H. Futerman

**Affiliations:** 1 Department of Biomolecular Sciences, Weizmann Institute of Science, Rehovot, 7610001 Israel; 2 Genetics and Molecular Pathology, SA Pathology at Women’s and Children’s Hospital and Adelaide Medical School, The University of Adelaide, Adelaide, SA 5005 Australia; 3 Department of Neurodegenerative Disease, UCL Dementia Research Institute, University College London, London, WC1N 3BG UK; 4 The Joseph Meyerhof Professor of Biochemistry at the Weizmann Institute of Science, Rehovot, Israel

**Keywords:** Metabolomics, Biochemistry

## Abstract

A number of genetic risk factors have been identified over the past decade for Parkinson’s Disease (PD), with variants in *GBA* prominent among them. *GBA* encodes the lysosomal enzyme that degrades the glycosphingolipid, glucosylceramide (GlcCer), with the activity of this enzyme defective in Gaucher disease. Based on the ill-defined relationship between glycosphingolipid metabolism and PD, we now analyze levels of various lipids by liquid chromatography/electrospray ionization-tandem mass spectrometry in four brain regions from age- and sex-matched patient samples, including idiopathic PD, PD patients with a *GBA* mutation and compare both to control brains (*n* = 21 for each group) obtained from individuals who died from a cause unrelated to PD. Of all the glycerolipids, sterols, and (glyco)sphingolipids (251 lipids in total), the only lipid class which showed significant differences were the gangliosides (sialic acid-containing complex glycosphingolipids), which were elevated in 3 of the 4 PD-GBA brain regions. There was no clear correlation between levels of individual gangliosides and the genetic variant in Gaucher disease [9 samples of severe (neuronopathic), 4 samples of mild (non-neuronopathic) *GBA* variants, and 8 samples with low ﻿pathogenicity variants which have a higher risk for development of PD]. Most brain regions, i.e. occipital cortex, cingulate gyrus, and striatum, did not show a statistically significant elevation of GlcCer in PD-GBA. Only one region, the middle temporal gyrus, showed a small, but significant elevation in GlcCer concentration in PD-GBA. We conclude that changes in ganglioside, but not in GlcCer levels, may contribute to the association between PD and *GBA* mutations.

## Introduction

The genetic association between genes related to lysosomal function and Parkinson’s disease (PD) is now well established^1^, suggesting that the lysosome may play a crucial role in PD pathogenesis^2^. In contrast, the mechanistic and biochemical association between lysosomal function and PD is unresolved and a matter of ongoing discussion^2–4^. Of the lysosomal genes, *GBA* is the most prominent, with 5–25% of PD patients^5^ carrying a *GBA* mutation. *GBA* encodes the lysosomal hydrolase acid β-glucosidase (GCase), the enzyme defective in the inherited metabolic disease, Gaucher disease^6,7^, in which two glycosphingolipid (GSL) substrates accumulate, namely glucosylceramide (GlcCer) and its deacetylated derivative, glucosylsphingosine (GlcSph)^8^. Gaucher disease is inherited in an autosomal recessive fashion, whereas most PD patients with *GBA* mutations (herein referred to as PD-GBA) are heterozygous *GBA* carriers.

A number of studies have investigated whether the mechanistic link between *GBA* and PD is related to altered levels of either GlcCer or GlcSph in the brain (reviewed in ref. ^9^). Many of these studies measured lipid levels in the substantia nigra, where loss of dopaminergic neurons occurs, and as a consequence interpretation of changes in GlcCer and GlcSph levels might be confounded by loss of cellular material due to loss of neurons rather than due to specific alterations in levels of a particular lipid class or species. To date, there is little evidence that GlcCer or GlcSph accumulate in brain samples from human PD or PD-GBA patients^9,10^, although some Lewy body disease patients with a *GBA* mutation show small but non-significant changes in GlcCer levels^11^. There is no evidence for GlcCer accumulation in the brains of heterozygous individuals with a *GBA* mutation, although there is a suggestion that GlcSph, but not GlcCer levels are elevated in the plasma of *GBA* carriers^12^, while a different analysis reported normal plasma GlcSph levels in *GBA* carriers^13^.

Despite the lack of evidence that the two direct substrates of GCase accumulate in *GBA* carriers or in PD-GBA patients, no systematic studies have been performed to determine whether levels of other GSLs in the same metabolic pathway might be altered, or whether levels of other brain lipids change in PD-GBA. This being the case, we have now systematically analyzed levels of the three major lipid classes found in the brain [i.e. glycerolipids, sphingolipids (SLs), and sterols] in a cohort of patient brain samples, which includes 21 idiopathic PD patients (IPD), 21 PD-GBA patients and 21 age and sex-matched controls. Levels of 251 individual lipid species were analyzed by liquid chromatography/electrospray ionization-tandem mass spectrometry (LC-ESI-MS/MS). Rather than focusing on the substantia nigra, we used 4 other brain regions, including the striatum (STR), which is part of the nigrostriatal pathway and thus directly impacted by dopamine loss from the substantia nigra, while the three other regions, the occipital cortex (OCC), middle temporal gyrus (MTG) and cingulate gyrus (CG) mainly receive dopaminergic input from the ventral tegmental area^14^ and are thus not directly affected by dopamine loss in the nigrostriatal pathway.

Changes in levels of lipids between the various patient groups were relatively modest. Interestingly, concentrations of gangliosides (i.e. sialic-acid containing glycosphingolipids) were elevated by a small but statistically significant amount in most brain areas from PD-GBA samples, in such a way that suggests upregulation of the pathway of ganglioside metabolism. Our data are consistent with our recent suggestion^9^ that cellular pathways, other than changes in GlcCer or GlcSph levels, need to be unearthed in order to delineate the mechanistic association between *GBA* mutations and PD, including up-stream GSLs in the catabolic pathway such as gangliosides.

## Results

### Quality analysis and brain distribution of lipid classes

**Fig. 1:**
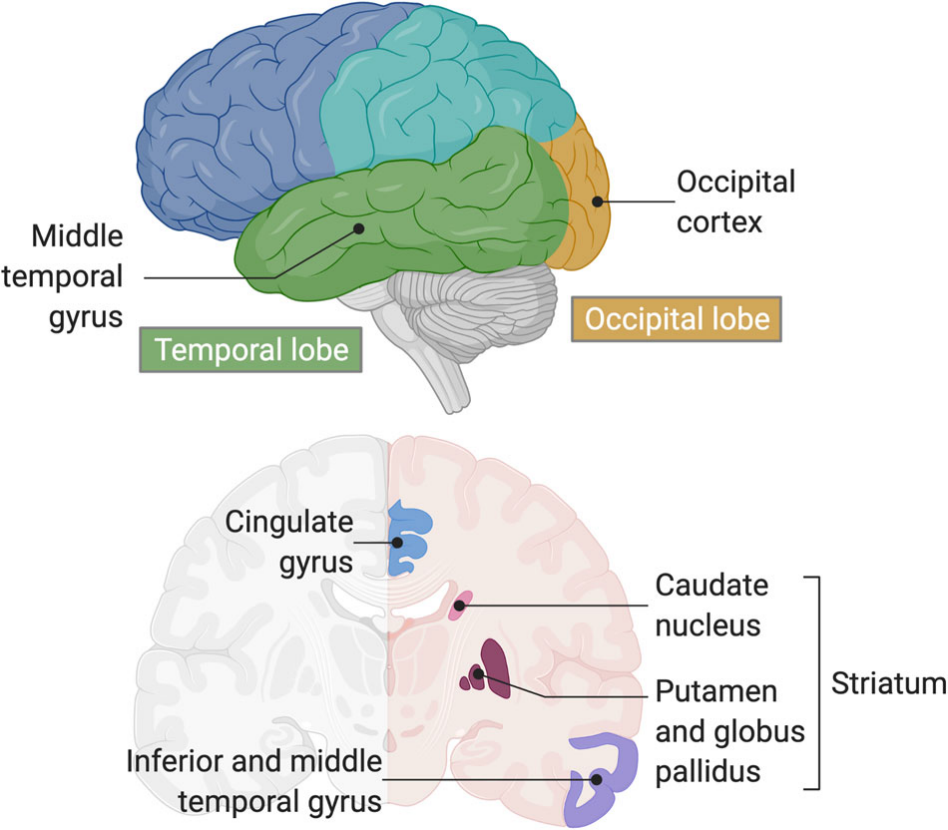
Brain regions used in the study. Schematic illustration of the brain regions used in this study. Created with BioRender.com.

**Fig. 2:**
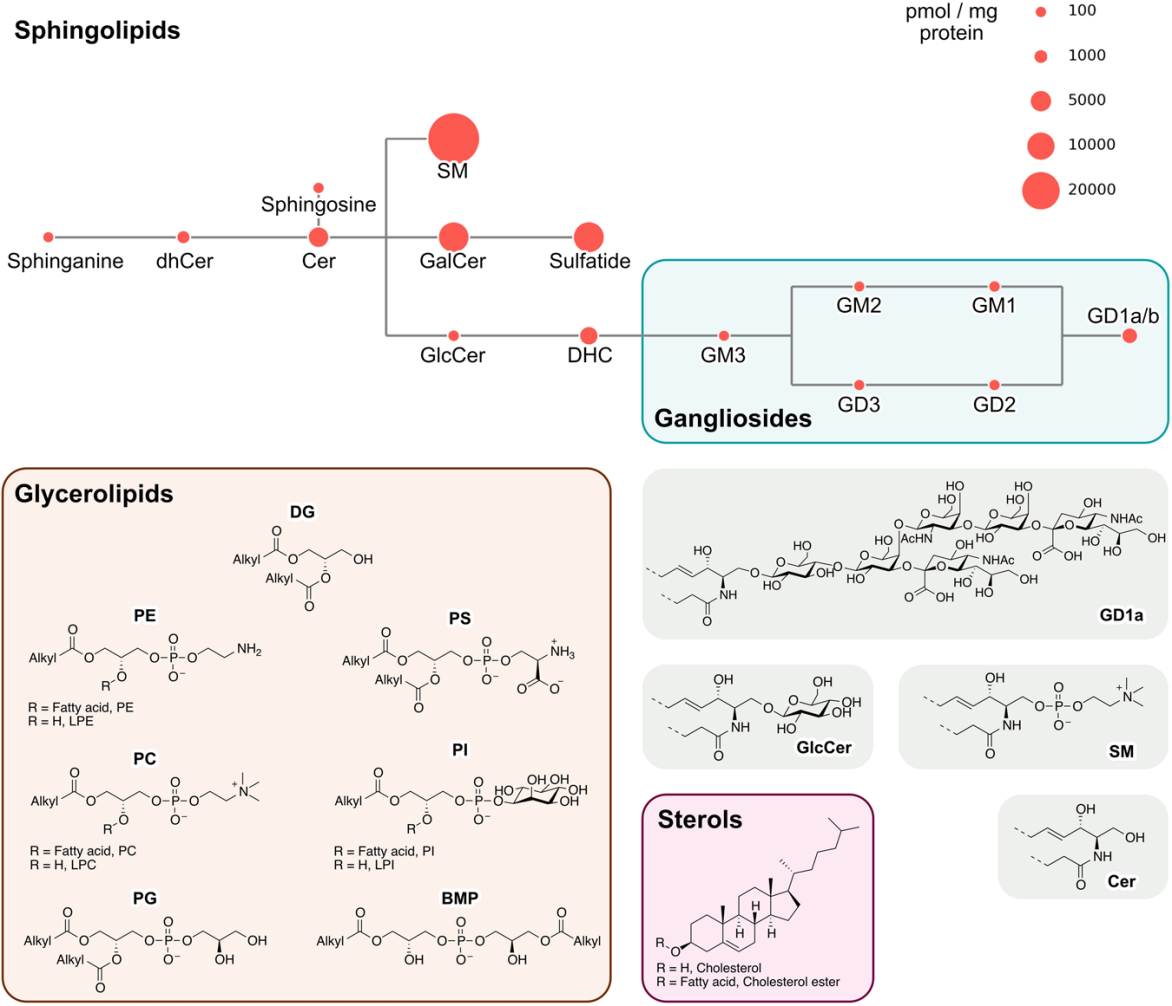
Lipid classes measured in the study. The upper portion of the figure shows the SLs that were measured, with the size of each circle (*red*) representing the average concentration (pmol/mg protein) of each lipid in control brains, averaged across three brain regions (MTG, CG, and STR). For instance, SM is the most abundant SL in the brain (37,695 pmol/mg protein) and gangliosides GD1a/b are the most abundant gangliosides (1803 pmol/mg protein). The lower panel shows the structures of some of the SLs, along with the structures of the glycerophospholipids and lysoglycerophospholipids, which vary between their head group, along with cholesterol and cholesterol ester. Abbreviations: CE cholesterol ester; DG diacylglycerol; PC phosphatidylcholine; PE phosphatidylethanolamine; PG phosphatidylglycerol; PI phosphatidylinositol; PS phosphatidylserine; LPC lysophosphatidylcholine; LPE lysophosphatidylethanolamine; LPI lysophosphatidylinositol; BMP bis(monoacylglycero)phosphate; Cer ceramide; dhCer dihydroceramide; SM sphingomyelin; GalCer galactosylceramide; GlcCer glucosylceramide; DHC dihexosylceramide.

Brain samples from four different brains regions (Fig. 1) were analyzed by LC-ESI-MS/MS. 251 lipid species were measured for most samples, including 6 sterols, 150 glycerophospholipids and lysoglycerophospholipids, and 95 SLs (Fig. 2). For the SL class, 4 simple SLs were measured (sphinganine, dihydroceramide, ceramide, and sphingosine), along with sphingomyelin (SM), sulfatide and a variety of gangliosides (sialic acid-containing GSLs), including GD1a/b, GD2, GD3, GM1, GM2, and GM3. The acyl chain distribution of all lipids was measured individually and also summed for each lipid class. The average levels of each of the SLs from 3 regions from control brain samples (Fig. 2) is consistent with published data on control human brains from elderly individuals, inasmuch as SM^15^ was the major SL, and gangliosides GD1a/b^16^ were the major gangliosides.

We first determined whether the age of the patients, their gender or the type of *GBA* variant influenced the lipid profile in each brain region by performing principal component analysis (PCA) on all lipid classes. No separation by group according to age, gender, or *GBA* mutation was detected, indicating that there is no correlation between lipid levels and age, gender, or *GBA* mutation (Supplementary Fig. 1).

To determine the difference in the distribution of the major lipid classes between the four brain regions, and more importantly for the current study, between the three different patient sample groups, total concentrations of lipid classes (expressed per mg of protein) were compared between control, IPD and PD-GBA samples using mixed analysis of variance (mixed ANOVA) (Table 1). As expected, a statistically-significant difference (*p* < 0.005) between the different brain regions was observed in concentrations of all lipid classes (with the exception of SM), consistent with studies showing that lipid classes are differentially distributed across different brain regions^17,18^. Analysis of lipid concentrations across the sample groups indicated that total levels of each lipid class did not differ between control, IPD, or PD-GBA samples (*p* > 0.1), with the exception of gangliosides (*p* < 0.001). Further analysis by mixed ANOVA indicated that the two factors, brain region, and sample group, independently affected ganglioside levels, indicating that changes in ganglioside levels are not restricted to one brain region.

**Table 1:** Effect of sample group and brain region on lipid levels.

Lipid	Effect	*F*_*(DF)*_	*p* value
GlcCer	Brain region	36.439 _(2.47, 101.29)_	<0.001
	Group	2.174 _(2, 41)_	0.127
	Group:brain region	0.358 _(4.94, 101.29)_	0.874
Gangliosides	Brain region	36.54 _(2.34, 107.63)_	<0.001
	Group	7.579 _(2, 46)_	0.001
	Group:brain region	0.325 _(4.68, 107.63)_	0.887
SM	Brain region	0.734 _(3, 132)_	0.534
	Group	1.6 _(2, 44)_	0.213
	Group:brain region	1.275 _(6, 132)_	0.273
Other SLs	Brain region	4.953 _(3, 138)_	0.003
	Group	0.886 _(2, 46)_	0.447
	Group:brain region	0.829 _(6, 138)_	0.684
Glycerolysophospholipids	Brain region	8.398 _(1.92, 88.47)_	<0.001
	Group	0.719 _(2, 46)_	0.493
	Group:brain region	1.528 _(3.85, 88.47)_	0.203
Glycerophospholipids	Brain region	5.459 _(1.12, 51.57)_	<0.001
	Group	1.292 _(2, 46)_	0.222
	Group:brain region	0.524 _(2.24, 51.57)_	0.339
Bis(monoacylglycero)phosphate	Brain region	16 _(3, 138)_	<0.001
	Group	1.07 _(2, 46)_	0.352
	Group:brain region	0.878 _(6, 138)_	0.513
Cholesteryl ester	Brain region	166.205 _(2.48, 109.02)_	<0.001
	Group	0.884 _(2, 44)_	0.42
	Group:brain region	0.744 _(4.96, 109.02)_	0.591

The effect of the brain region (*Brain region*) and sample group (*Group*), i.e. control, IPD, or PD-GBA on lipid levels, and the interaction between them (*Group:brain region*) was analyzed by mixed ANOVA. *F* statistic, degrees of freedom (DF) and *p* value are reported. For instance, GlcCer levels differ significantly between brain regions (*F*_2.47,101.29_ = 36.439, *p* < 0.001) whereas SM levels are similar between regions (*F*_3,132_ = 0.734, *p* = 0.534). Gangliosides were the only lipid class to change between sample groups (*F*_2,46_ = 7.579, *p* = 0.001) irrespective of brain region (*F*_4.68,107.63_ = 0.325, *p* = 0.887).

**Fig. 3:**
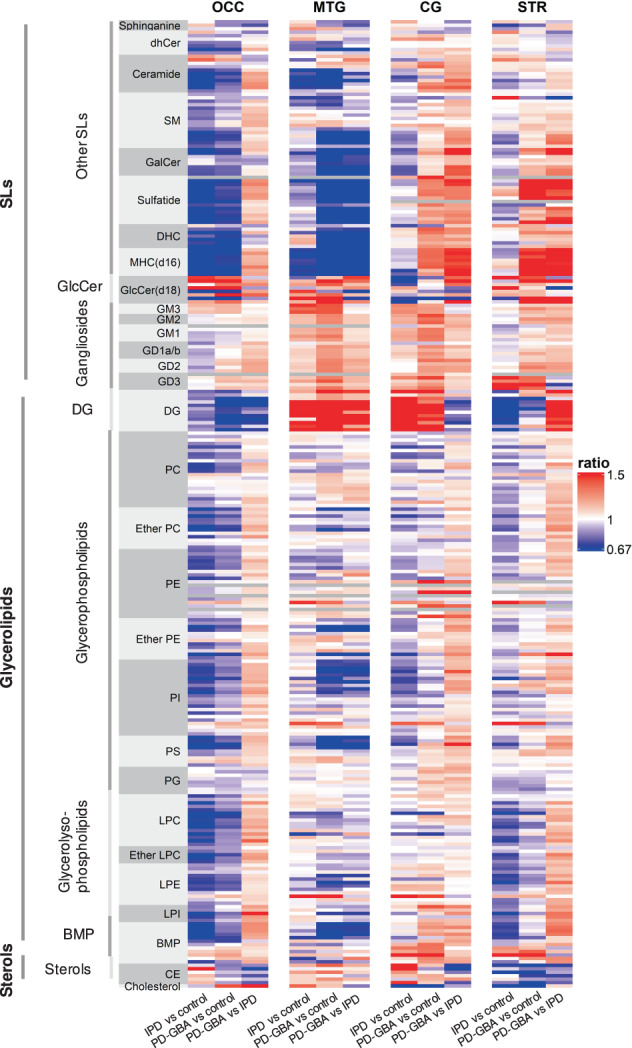
Lipid concentrations in IPD and PD-GBA compared to control brain samples. Data are shown as a heatmap for each individual lipid class in each brain region, with each of the 251 rows corresponding to a lipid species with different chain lengths. Ratios of lipid concentrations are shown for IPD *versus* control, PD-GBA *versus* control, and PD-GBA *versus* IPD. *Blue* indicates a ratio of <1 and *red* a ratio of >1, as indicated in the key; *grey* indicates not detected.

To determine changes in concentrations of individual lipids, data was interrogated by ANOVA followed by post-hoc pairwise multiple comparisons using the Tukey’s honestly significant difference test (Tukey HSD). Data was plotted as a heatmap to give an overview of changes in lipid concentrations between IPD samples compared to controls, between PD-GBA and controls, and between PD-GBA and IPD. With the exception of gangliosides, no consistent and overriding pattern of change was detected between the 4 brain regions or between the sample groups (Fig. 3), indicating that any changes in lipid concentrations are brain region-dependent. One of the most noticeable changes was that concentrations of some lipids were reduced in both IPD and PD-GBA samples in the OCC, with a somewhat greater reduction in IPD. More specifically, concentrations of C32-C38-phosphatidylethanolamine (PE) (Supplementary Fig. 2a), C36:1- and C37:1-SM (Supplementary Fig. 2b), lysophosphatidylcholine (LPC) (Supplementary Fig. 2c) including ether LPC (Supplementary Table 1), C18:2- and C20:3-lysophosphatidylethanolamine (LPE) (Supplementary Fig. 2d), lysophosphatidylinositol (LPI) (Supplementary Fig. 2e), phosphatidylinositol (PI) (Supplementary Table 1), phosphatidylcholine (PC) including ether PC (Supplementary Table 1), C23:0-, C24:0- and C25:0-ceramide (Supplementary Table 1) and C35:1-, C36:1- and C37:1-sphingomyelin (SM) (Supplementary Table 1) were reduced mostly in IPD but not in PD-GBA in the OCC.

### Analysis of glycosphingolipid levels

**Fig. 4:**
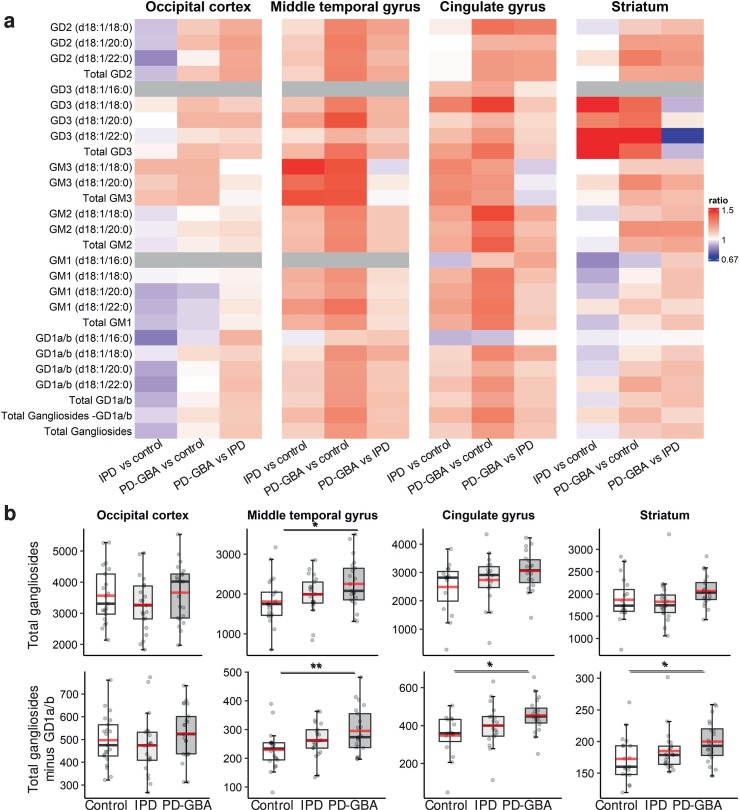
Ganglioside concentrations in IPD and PD-GBA brain. **a** Heatmap displaying ratio of concentrations of ganglioside species in the four different brain regions, along with the ratio of their total concentrations (i.e. the sum of all individual species). *Blue* indicates a ratio of <1 and *red* a ratio of >1; *grey* indicates not detected. **b** Boxplots of total ganglioside concentrations (pmol/mg protein) with (*upper* panel) and without (*lower* panel) GD1a/b (major gangliosides in human brain, see Fig. 2). The box represents lower quartile, median and upper quartile (*black*). The whiskers represent the minimum and maximum values, up to 1.5 times the interquartile range from the bottom or the top of the box to the furthest data point within that distance, thus excluding outliers. The mean is shown in *red*. *p ≤ 0.05; **p ≤ 0.01; ***p ≤ 0.001.

**Fig. 5:**
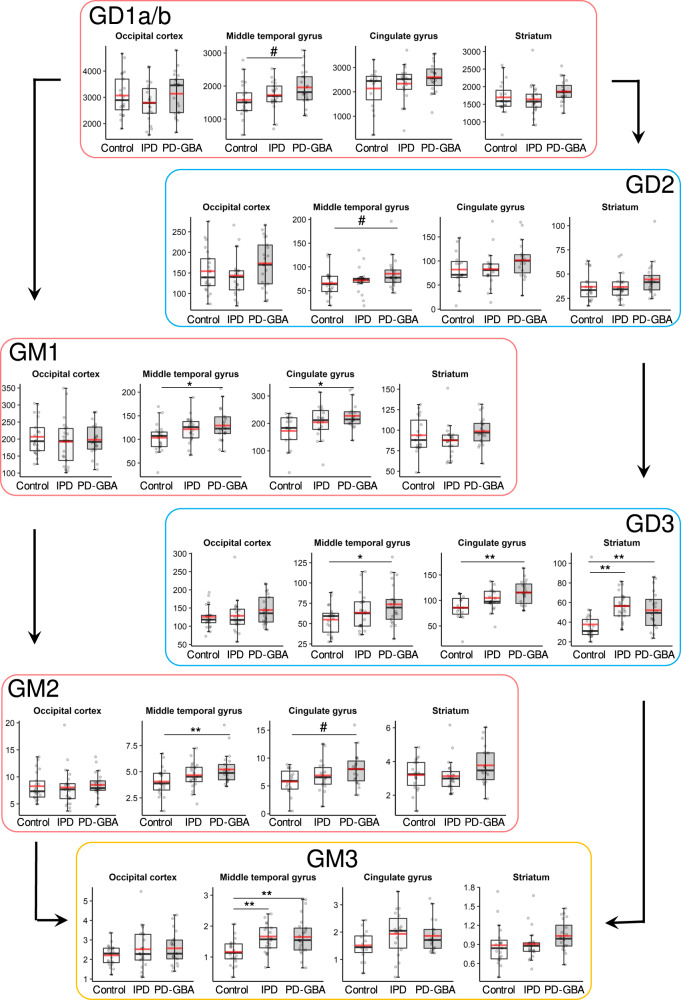
Individual ganglioside concentrations in IPD and PD-GBA brain. Boxplots of total ganglioside concentrations (pmol/mg protein) expressed according to the order of their lysosomal degradation. The box represents lower quartile, median, and upper quartile (*black*). The whiskers represent the minimum and maximum values, up to 1.5 times the interquartile range from the bottom or the top of the box to the furthest data point within that distance, thus excluding outliers. The mean is in *red*. ^#^*p* ≤ 0.1 *p ≤ 0.05; **p ≤ 0.01; ***p ≤ 0.001.

The most consistent change, as indicated in Table 1, was in ganglioside concentrations, which were increased in a number of brain areas in PD-GBA samples, and to a smaller extent in IPD, such that in almost all cases, the ratio of ganglioside concentration was higher in PD-GBA compared to IPD (Fig. 4). This was most noticeable in the MTG, where the mean concentration of all gangliosides was 2251 ± 611 pmol/mg protein in PD-GBA compared to 1984 ± 491 in IPD and 1808 ± 579 in controls (Fig. 4b) (*p* < 0.05 for PD-GBA *versus* control). A similar result, and with a higher degree of statistical significance, was obtained when total ganglioside concentrations were analyzed, but excluding the most abundant gangliosides, namely GD1a/b (which were found at ~10-fold higher levels than the other gangliosides) (Fig. 4b). While statistical significance was not reached for total gangliosides in all brain areas, perhaps due to sample size and variability between individuals, a clear and consistent pattern can be seen indicating that total ganglioside concentrations are higher in PD-GBA compared to IPD and control (Figs. 4 and 5). A statistically significant elevation was observed for GM1 in the MTG and CG, GM2 in the MTG, GM3 in the MTG, and GD3 in the MTG, CG and STR. This pattern of elevation in the PD-GBA group extends to all other brain regions for all gangliosides measured, with the exception of GM1 in the OCC (Fig. 5). Also, levels of some individual ganglioside species are also higher, as documented for GD1a/b (Supplementary Fig. 3), GD2 (Supplementary Fig. 4), GD3 (Supplementary Fig. 5), GM1 (Supplementary Fig. 6) and GM2 (Supplementary Fig. 7).

**Fig. 6:**
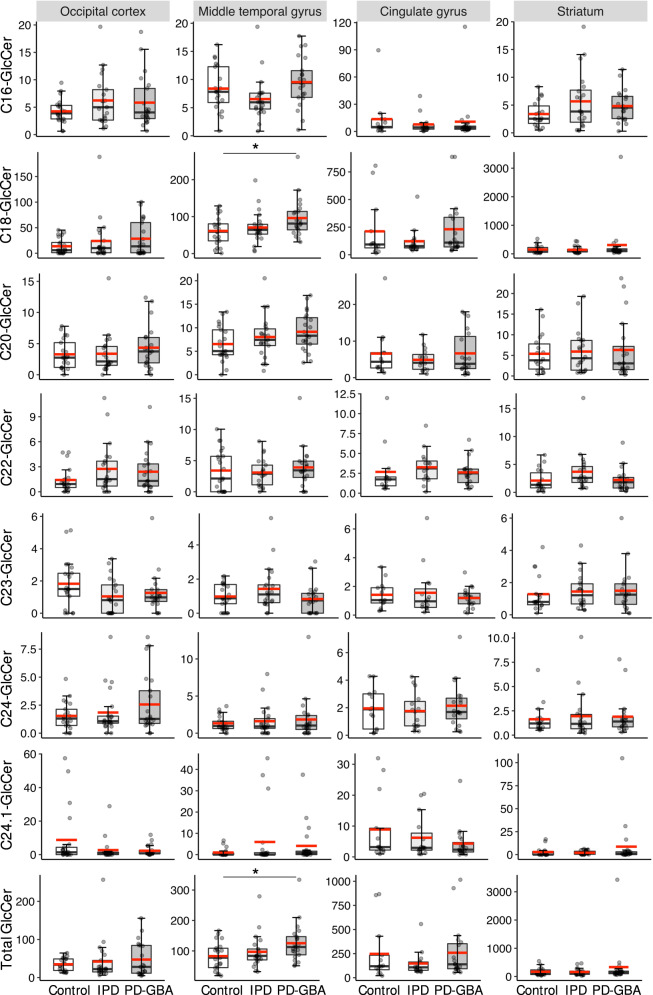
GlcCer concentrations in IPD and PD-GBA brain. Boxplots of concentrations of individual GlcCer species (pmol/mg protein) along with the sum of all species (Total). The box represents lower quartile, median and upper quartile (*black*). The whiskers represent the minimum and maximum values, up to 1.5 times the interquartile range from the bottom or the top of the box to the furthest data point within that distance, thus excluding outliers. No outliers were removed from the data, including sample PG6 which had concentrations of C18-GlcCer ~10-fold higher than most other samples in the striatum of PD-GBA; there is no experimental justification for removing this sample from the analysis although it is assumed to be due to an unidentified technical issue since concentrations of other lipids were not abnormal for PG6 in other brain regions. The mean is in *red*. *p ≤ 0.05; **p ≤ 0.01; ***p ≤ 0.001.

In contrast, no changes in concentrations of GlcCer species was detected in any of the brain regions, with the exception of the MTG, where C18-GlcCer was higher in PD-GBA than in control samples (1.57-fold; *p* < 0.05) as well as the total concentration of GlcCer (1.51-fold; *p* < 0.01), which reflects C18-GlcCer levels since this is the most abundant GlcCer species (~10–100 fold higher than other GlcCer species) (Fig. 6). In some cases a trend towards elevated GlcCer concentrations in PD-GBA was observed, without reaching statistical significance, i.e. C18-, C20-, C24-GlcCer and total GlcCer in the OCC, C16-, C20-, C22- and C24-GlcCer in the MTG and C18-, C20-, C23-, C24:1-GlcCer and total GlcCer in the STR (Fig. 6). However, even if statistical significance was reached, the extent of elevation of GlcCer was relatively small, suggesting that GlcCer is unlikely to play a critical role in the pathophysiology of PD-GBA^9^.

**Fig. 7:**
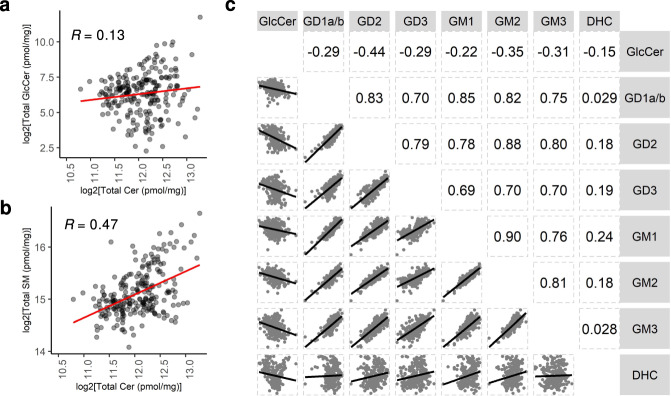
Correlation between GlcCer, gangliosides, and ceramide. Matrices were generated using combined data from all four brain regions and all sample groups (i.e. control, IPD, and PD-GBA). Pearson correlation matrices of **a** GlcCer *versus* ceramide, **b** ceramide *versus* SM, and **c** GlcCer, and DHC *versus* total gangliosides. Correlation coefficient values are indicated. Axes represent log_2_ scale of lipid concentrations.

We next determined whether there is a correlation between ganglioside and GlcCer or ceramide concentrations in individual samples, since all three are components of the GSL metabolic pathway. No correlation was detected between concentrations of GlcCer and ceramide (Fig. 7a), probably because ceramide can be generated via a number of different metabolic pathways^19^, but a low correlation was found between ceramide and SM (Fig. 7b, *R* = 0.47). Likewise, no correlation was found between GlcCer and concentrations of individual gangliosides, although a small negative correlation was observed, such that higher concentrations of gangliosides resulted in lower levels of GlcCer (Fig. 7c, *R* = −0.22 to −0.44). However, a high correlation was detected between individual gangliosides (Fig. 7c, *R* = 0.70–0.90). For instance, a high concentration of GD1a/b correlated with a high concentration of GM1 and GM2, indicating that the pathway of ganglioside metabolism is upregulated rather than only changes in levels of individual gangliosides. No correlation was found between concentration of dihexosylceramide (DHC) and individual gangliosides (Fig. 7c, *R* = 0.028–0.24).

**Fig. 8:**
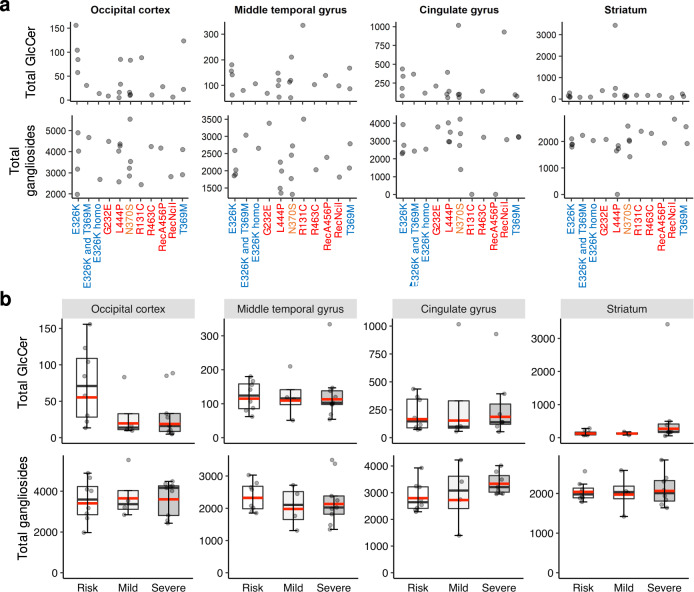
Correlations between GlcCer and ganglioside concentrations and the *GBA* variant. **a** GlcCer (upper panel) and total ganglioside (lower panel) concentrations shown according to the different *GBA* mutations [severe mutations, i.e. G232E, R131C, L444P, R463C, RecA456P, RecNciI (*red*), mild mutations, i.e. N370S (*orange*) and risk factors E326K, T369M (*blue*)]. Each point indicates GlcCer or ganglioside concentrations in an individual patient. **b** Boxplots of total GlcCer (*upper* panel) and total ganglioside concentrations (*lower* panel) in the PD-GBA group according to mutation severity. The box represents lower quartile, median, and upper quartile (*black*). The whiskers represent the minimum and maximum values, up to 1.5 times the interquartile range from the bottom or the top of the box to the furthest data point within that distance, thus excluding outliers. The mean is in *red*. No statistical significance was reached using the t-test.

Finally, we analyzed whether there is a correlation between GlcCer and ganglioside concentrations and the type of *GBA* mutation in the PD-GBA samples. *GBA* mutations were divided into three groups according to the mutation severity in Gaucher disease, with severe mutations defined as G232E, R131C, L444P, R463C, RecA456P, RecNciI, mild mutations defined as N370S and risk factors defined as E326K, T369M^20–22^. No correlation was detected between the *GBA* mutation/mutation severity and GlcCer or ganglioside concentrations (Fig. 8). Thus, GlcCer concentrations in the OCC of the four PD-GBA patients carrying the E326K mutation ranged between ~50 and ~150 pmol/mg protein (Fig. 8a). GlcCer concentrations in the MTG of the four PD-GBA patients carrying the N370S mutation ranged between ~0 and ~200 pmol/mg protein. Similarly, total ganglioside concentrations in the OCC of the four PD-GBA patients carrying the E326K mutation ranged between ~2000 and ~5000 pmol/mg protein (Fig. 8a). Furthermore, there is no statistical difference in the concentrations of GlcCer and gangliosides between mild and severe mutations (Fig. 8b).

## Discussion

The most notable finding of the current study is that the ganglioside concentrations were increased in most of the brain areas studied, in particular in PD-GBA, in a manner that is consistent with upregulation of the pathway of ganglioside metabolism rather than increases in levels of individual ganglioside species. In principle, changes in levels of gangliosides could come from either changes in their rate of biosynthesis, or from changes in their rate of degradation^23^, although we cannot formally distinguish between these two possibilities. There were no significant differences in most of the other lipids measured in the four human brain regions between samples from control, PD, and PD-GBA patients, and moreover, concentrations of the lipid substrate of GCase were essentially unchanged, consistent with most studies^9^.

One possible criticism of our study is that we did not analyze lipids in the main tissue affected in PD, namely the substantia nigra. The reason for this was two-fold. First, little tissue was available from the substantia nigra due to its obliteration during the progression of PD. Second, analysis of lipid concentrations in the substantia nigra would likely reflect tissue loss rather than loss of specific lipid species. Thus, we chose tissues that are less obviously affected by neuronal loss (with the exception of the STR, which is directly affected by axonal loss from the substantia nigra), but are nevertheless impacted by PD pathology, such as the OCC, MTG, and CG, which develop α-synuclein pathology at different stages of the disease^24^ and are involved in the manifestation of non-motor symptoms^25^.

There are very few studies, if any, with which to compare our data, although some recent studies did measure gangliosides in the brain of PD patients, and demonstrated that ganglioside levels were lower in the substantia nigra of IPD patients compared to controls^26,27^. These differences may be explained by the fact that both studies^26,27^, used a different brain region than used in our study. Since we have shown that lipid distribution differs between brain regions (Table 1), it is not unreasonable to suggest that the SN also has a different lipid composition and, therefore would show a different lipid pattern than detected in our study, which did not include the SN. In addition, since gangliosides are highly expressed in neurons, reduction of gangliosides in the SN could conceivably be explained by the neuronal death in this region. Furthermore, differences may also be attributed to the different methodologies used in the studies, whereby we used LC-ESI-MS/MS and not ﻿High-Performance Thin-Layer Chromatography or high-performance liquid chromatography.

In another study, GM1 and GD1a ganglioside levels were reduced in gray matter from the OCC of IPD patients, although this study used a relatively small sample size^28^. Levels of C18-GM2 and GM3 were not affected in the putamen or cerebellum in IPD or PD-GBA, although a non-statistical trend of elevation was seen in the putamen of PD-GBA patients^29^. GM3 levels were elevated in the motor cortex of patients with Lewy body disorders carrying a *GBA* mutation, but not in patients with Lewy body disorders which did not carry a *GBA* mutation^11^. Finally, levels of several gangliosides were reduced in the cerebrospinal fluid of IPD samples with the exception of GA2 and GM1a, which were unchanged, and of ganglioside GM3 which was elevated^26^. The latter is important, since GM3 is not normally present in the mature brain, and elevation of GM3 levels might be related to a survival mechanism based on the key role that GM3 plays in neurodevelopment^30^. Elevated GM3 in the cerebrospinal fluid may also serve as a biomarker for disease severity^31^. Elevation of brain gangliosides have also been reported in other neurodegenerative conditions, such as Alzheimer’s disease^32,33^ and in several lysosomal storage diseases^34–36^ (reviewed in ref. ^37^).

It is difficult to compare studies from different tissues and different patient cohorts, but our systematic study suggests that, with one or two exceptions, ganglioside concentrations are elevated to a small extent in the IPD group, but elevated to a larger and statistically significant extent in the PD-GBA group, suggesting that gangliosides may play a specific role in the pathophysiology of PD-GBA. This is perhaps not surprising since gangliosides are enriched in the brain, and specifically in neurons^23^. The precise role of gangliosides have proven difficult to define, but in general, they are involved in processes ranging from modulation of membrane properties to modulation of apoptosis, autophagy, and neurite outgrowth^23^. In addition, ganglioside GM1 may have neurotrophic and neuroprotective properties, and modulation of GM1 levels was suggested as a treatment strategy for PD, although this approach has not been successful^38^. Moreover, the addition of exosomes containing gangliosides may accelerate α-synuclein aggregation^39,40^. The latter may suggest that the long-term elevation of ganglioside levels in PD-GBA reported in our study may exacerbate α-synuclein pathology and/or cell death-related alterations^41–43^, or potentially be part of the pathway by which the lysosome becomes overloaded, although a well-defined rationale for a role of gangliosides, rather than other lipids in lysosomal overload, is difficult to pin down. Interestingly, gangliosides, specifically GM1 and GM3, have been implied in amyloid pathology in Alzheimer’s disease^44^.

Although a number of attempts have been made to measure whether GlcCer levels change in PD-GBA, most have reported little or no change (reviewed in ref. ^9^), consistent with our current data. In retrospect, this is perhaps not surprising since there is no evidence that *GBA* carriers (i.e. heterozygotes) display Gaucher disease symptoms which would reflect changes in GlcCer levels, and there is, therefore, no reason to assume that PD patients with a heterozygous *GBA* mutation would present with elevated GlcCer levels. We appreciate that our sample size was perhaps not large enough to detect a very small change in GlcCer levels in PD-GBA, and that in some cases a non-significant trend towards an increase was observed. However, even if increasing the sample size were to lead to a statistically-significant increase in GlcCer in PD-GBA patients, the question remains as to whether this change would be able to influence cellular pathology. Indeed, in Gaucher disease, GlcCer concentrations increase by at least ~10-fold^8^, significantly higher than the small changes occasionally observed in our study. Moreover, basal GlcCer concentrations are in the region of 50–200 pmol/mg protein whereas ganglioside concentrations are ~10-fold higher (2000–5000 pmol/mg protein), with the latter increasing by ~400 pmol/mg protein in PD-GBA versus control samples. One limitation of our study is that we did not measure GlcSph, which accumulates as a result of *GBA* mutations in Gaucher disease^8^. However, since GlcCer accumulates to a much higher extent than GlcSph in Gaucher disease, changes in GlcSph levels in PD-GBA are unlikely to be significant.

In summary, our data reveal a change in ganglioside levels in non-dopaminergic regions of the human brain in PD-GBA, and very few changes in other lipid classes, including GlcCer. This clearly has implications for therapeutic approaches that might target the GCase/GlcCer/GlcSph axis. It may, however, suggest that one or other aspect of ganglioside metabolism might be a suitable therapeutic target for PD-GBA patients.

## Methods

### Human brain samples

Human brain samples were obtained as frozen tissue from the Queens Square Brain Bank of London, and include 21 idiopathic PD patients without a *GBA* mutation (IPD), 21 PD-GBA patients and 21 controls, from four different brain regions (OCC, MTG, CG, and STR). The CG region contained only 52 samples (15 controls, 18 IPD, and 19 PD-GBA), and the STR region contained 60 samples (19 controls, 21 IPD and 20 PD-GBA), for a total of 238 samples. The groups in this study were age- and sex-matched. *GBA* was sequenced by the Queens Square Brain Bank of London and *GBA* mutations for each of the PD-GBA patients are documented (Table 2). The study was approved by the National Research Ethics Service (NRES) committee, London (127366). All participants gave written informed consent.

**Table 2:** Samples used in the study.

Sample ID	Group	Gender	Age at death	*GBA* mutation
C1	Control	M	81	
C2	Control	M	95	
C3	Control	M	87	
C4	Control	M	63	
C5	Control	F	64	
C6	Control	F	53	
C7	Control	F	85	
C8	Control	F	56	
C9	Control	F	86	
C10	Control	F	68	
C11	Control	M	43	
C12	Control	M	71	
C13	Control	F	87	
C14	Control	F	83	
C15	Control	F	80	
C16	Control	M	89	
C17	Control	F	92	
C18	Control	M	87	
C19	Control	F	78	
C20	Control	M	88	
C21	Control	M	84	
PD1	IPD	F	55	
PD2	IPD	M	67	
PD3	IPD	M	66	
PD4	IPD	F	61	
PD5	IPD	M	91	
PD6	IPD	M	85	
PD7	IPD	M	65	
PD8	IPD	M	68	
PD9	IPD	M	75	
PD10	IPD	M	63	
PD11	IPD	M	59	
PD12	IPD	M	81	
PD13	IPD	M	81	
PD14	IPD	M	81	
PD15	IPD	M	92	
PD16	IPD	M	82	
PD17	IPD	F	83	
PD18	IPD	M	80	
PD19	IPD	M	79	
PD20	IPD	F	73	
PD21	IPD	M	83	
PG1	PD-GBA	M	62	G232E
PG2	PD-GBA	M	55	R131C
PG3	PD-GBA	F	67	L444P
PG4	PD-GBA	M	91	N370S
PG5	PD-GBA	M	85	N370S
PG6	PD-GBA	M	65	L444P
PG7	PD-GBA	M	68	R463C
PG8	PD-GBA	M	57	RecA456P
PG9	PD-GBA	F	64	RecNciI
PG10	PD-GBA	M	59	L444P
PG11	PD-GBA	F	57	L444P
PG12	PD-GBA	M	82	N370S
PG13	PD-GBA	M	83	E326K
PG14	PD-GBA	F	85	E326K and T369M
PG15	PD-GBA	M	84	E326K
PG16	PD-GBA	M	83	T369M
PG17	PD-GBA	M	81	N370S
PG18	PD-GBA	F	80	T369M
PG19	PD-GBA	M	79	E326K
PG20	PD-GBA	F	78	E326K Homo
PG21	PD-GBA	F	79	E326K

Age, gender (M, male; F, female), and GBA mutations are shown. Tissues from 4 brain regions were available for most of the samples.

### Quantification of lipids by mass spectrometry

Lipid extracts were prepared from frozen tissue samples of the CG, MTG, OCC and STR as described^34^ with addition of 10 pmol each of bis(monomyristoylglycero)phosphate (BMP(14:0/14:0)), 1-myristoyl-2-hydroxy-sn-glycero-3-phosphoethanolamine (PE(17:0/17:0)), and 1,2 diheptadecanoyl-sn-glycero-3[phospho-L-serine] (PS(17:0/17:0)); 1 pmol each of D-*erythro*-sphinganine (C17 base) (Sph(d17:0)) and 1,2-diheptadecanoyl-sn-glycero-3[phospho-rac-(1-glycerol)] (PG(17:0/17:0)); 5 pmol 1,2-ditridecanoyl-sn-glycero-3-phosphocholine (PC(13:0/13:0)); 100 pmol of cholesteryl-2,2,3,4,4,6-d6 octadecanoate (CE 18:0 *d6*); and 20 pmol of 1,3-dipentadecanoin (DG(15:0/15:0)) as internal standards. With the exception of CE 18:0 d6 (CDN Isotopes, Quebec, Canada) and DG(15:0/15:0) (Sigma Aldrich, St. Louis, MI), all lipid standards were purchased from Avanti Polar Lipids (Alabaster, AL). Lipid extracts were analysed by liquid chromatography electrospray ionisation tandem mass spectrometry (LC-ESI-MS/MS) with gangliosides quantified as in ref. ^45^, with the GD1a and 1b isoforms indistinguishable and therefore measured together; the remaining SLs were quantified as described in ref. ^34^. Total lipids for each class were determined by summing the concentrations of the individual species (see Supplementary Table 1 for the raw Lipidomics data).

For quantitation of GlcCer and its stereoisomer, galactosylceramide (GalCer), Agilent Bond Elut Lipid Extraction 96-well plates (Agilent Technologies, Santa Clara, CA) were utilised with lipid extraction^46^. Brain homogenates (0.1 mg protein) were prepared in up to 0.1 mL of 0.9% saline. Samples were spiked with 10 pmol each of N-palmitoyl-d3-glucocerebroside (GlcCer(d18:1/16:0 *d3*)) and N-pentadecanoyl-psychosine (GalCer(d18:1/15:0)) (Matreya LLC, State College, PA) as internal standards. Protein was precipitated by the addition of 0.9 mL of ice-cold CH_3_CN:CH_3_OH (99:1). Samples were vortexed for 30 s and sonicated in a water bath for 14 min at room temperature, and then briefly vortexed again and transferred into a well of the extraction plate and eluted by gravity. This was followed by the addition of 2 ×1 mL CH_3_CN:H_2_O (9:1). Sorbent beds were dried under low vacuum pressure prior to elution of lipids with 2 ×1 mL CHCl_3_:CH_3_OH (1:1). The eluent was dried under N_2_ at 40 °C. Prior to LC-ESI-MS/MS, samples were reconstituted in 0.2 mL of the mobile phase, transferred to a v-bottom 96-well plate and heat-sealed with foil.

Separation of GlcCer and GalCer was achieved on a Phenomenex Omega SUGAR column (3 µm; 150 × 2.1 mm) maintained at 30 °C with an Agilent 1290 inline filter containing a 0.3 µm frit placed in front of the column. The sample (4 µL) was injected into an isocratic mobile phase of CH_3_CN/H_2_O/CH_3_OH/CHOOH (95.5/2.5/2/0.5) and 5 mM NH_4_COOH, maintained at a flow rate of 0.7 mL/min. This was directed into the ESI source (ES 5500 V; ion source temperature 250 °C) of a SCIEX QTRAP 6500 tandem mass spectrometer in positive ion mode. Nitrogen (25 units) was used for the curtain gas; collision gas, medium; nebulizer gas 1, 20 units; auxillary gas 2, 40 units. The runtime for each sample was 5 min, and species were identified by MRM with GlcCer eluting approximately 0.1 min prior to GalCer. Authentic GlcCer(d18:1/16:0) and GalCer(d18:1/16:0) standards together with archived brain lipid extracts from a neuronopathic mouse model of Gaucher disease^47^ were used to aid in analyte identification. Individual lipids were quantified by relating the peak area to that of the corresponding internal standard using MultiQuant software (SCIEX, v. 3.0.1).

### Statistical analysis

Seven datasets, comprising each lipid class, were analyzed using R version 3.6.1. Principal Component Analysis (PCA) was used to analyze the influence of various factors on the data (age, gender, and *GBA* mutation). For statistical analyses, data was transformed to a log_2_ scale (zero values were replaced with 0.001 and non-available (NA) data were removed). The mixed analysis of variance (mixed ANOVA), ‘rstatix’ package, was used to compare different lipid groups versus brain regions and samples. Heatmaps were created using the ‘ComplexHeatmap’ package. Statistical significance was evaluated by ANOVA, followed by post-hoc pairwise comparisons using the Tukey’s honest significant difference test (Tukey HSD). Boxplots were created using the ‘ggplot2’ package. Pearson Correlation matrices were created using the ‘GGally’ package.

## Supplementary information

Supplemental Material

Data Set 1

## Data Availability

The authors declare that all data supporting the findings of this study are available within the paper and the supplementary information files.
