# Supplementary material for: Elevation of gangliosides in four brain regions from Parkinson’s disease patients with a GBA mutation
Source: NPJ Parkinsons Dis. 2022 Aug 6;8:99. doi: 10.1038/s41531-022-00363-2 (PMC9357011; doi:10.1038/s41531-022-00363-2)

**Supplementary Material**

**Supplementary Table 1.** See attached excel file which contains three sheets: 1) Ratio and statistical significance for all lipids; 2) the means of the concentrations of the lipids in pmol/mg protein; and 3) the raw data in pmol/mg protein.

**Supplementary Figure 1. Correlation of lipidomics data with age, gender and *GBA* mutation.** Lipidomics was performed on four brain regions (OCC, MTG, CG and STR) of control, IPD and PD-GBA (see Table 1 for a list of samples used). Representative data from the STR is shown, with total ganglioside and SL levels (excluding gangliosides) plotted *versus* age, gender or *GBA* mutation. No separation into groups is seen, and no separation was seen in any of the other brain regions (not shown). Controls, *circles*; IPD, *triangles*; PD-GBA, *squares*.

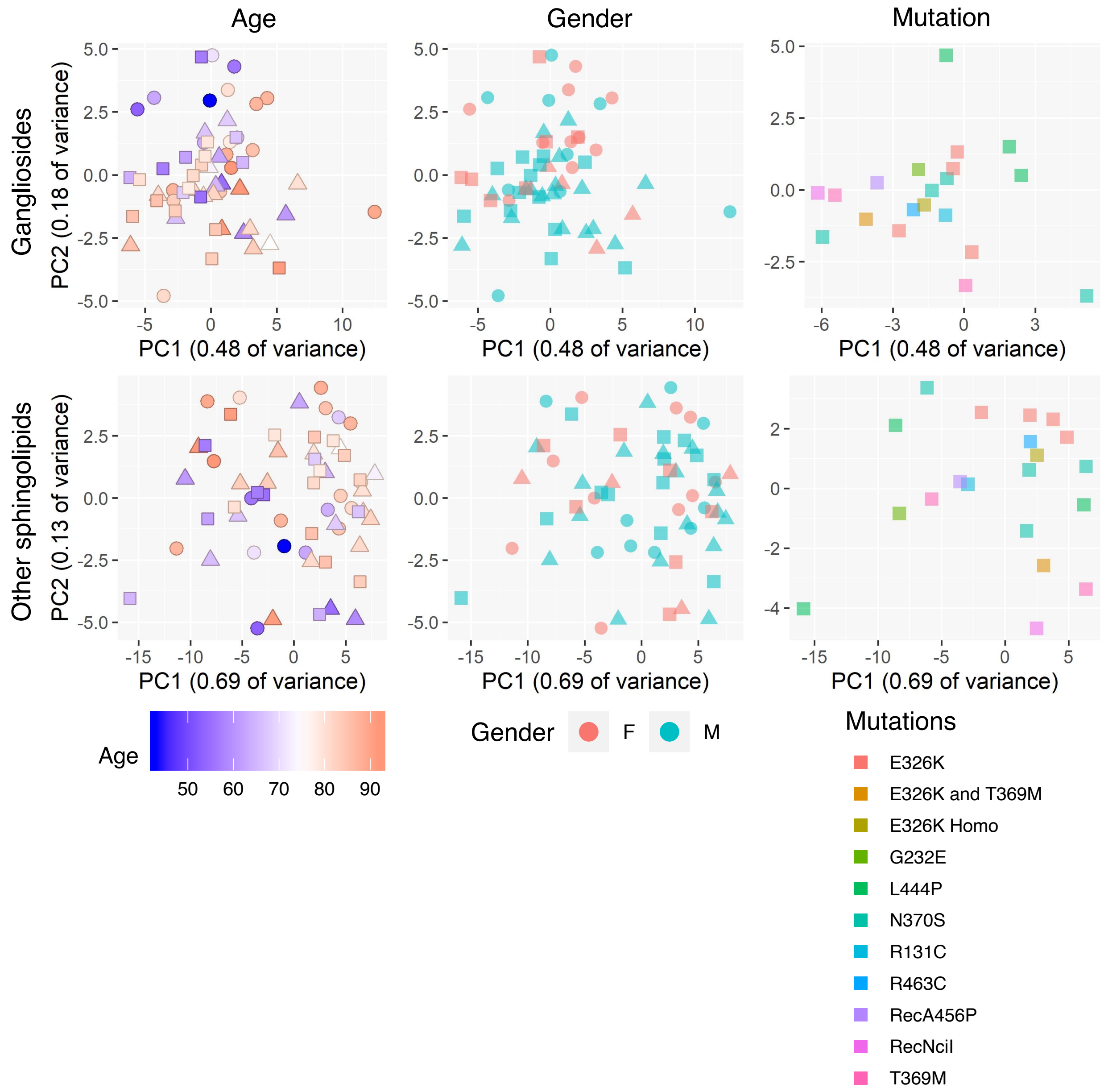

**Supplementary Figure 2. Lipid concentrations in the OCC of IPD patients.** Boxplots of the concentrations (pmol/mg protein) of significantly reduced lipids in the OCC of the IPD group. *a*, phosphatidylethanolamine (PE), *b*, sphingomyelin (SM), *c*, lysophosphatidylcholine (LPC), *d*, lysophosphatidylethanolamine (LPE), and *e*, lysophosphatidylinositol (LPI). The box represents lower quartile, median and upper quartile (*black*). The whiskers represent the minimum and maximum values, up to 1.5 times the interquartile range from the bottom or the top of the box to the furthest data point within that distance, thus excluding outliers. The mean is shown in *red*. *$p \leq 0.05$; **$p \leq 0.01$; ***$p \leq 0.001$.

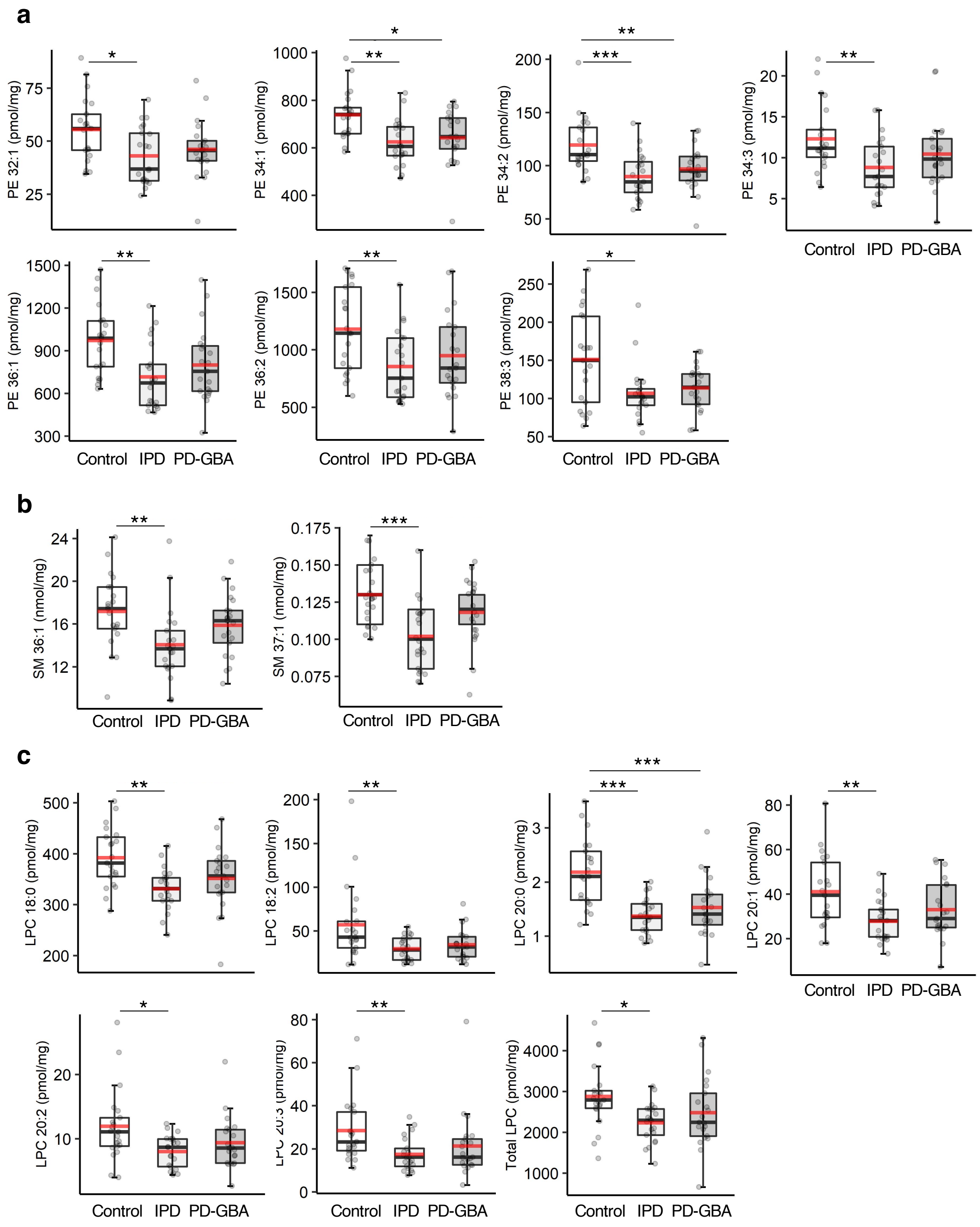

**d**

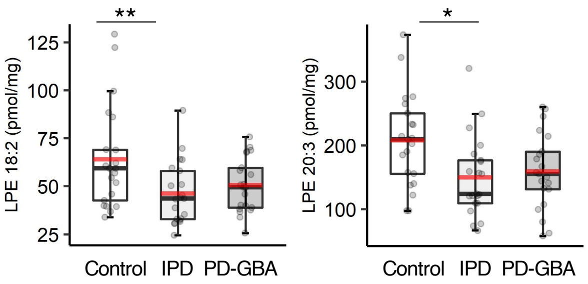
**
*
LPE 18:2 (pmol/mg)
125
100
75
50
25
LPE 20:3 (pmol/mg)
300
200
100
Control
IPD
PD-GBA
Control
IPD
PD-GBA

**e**

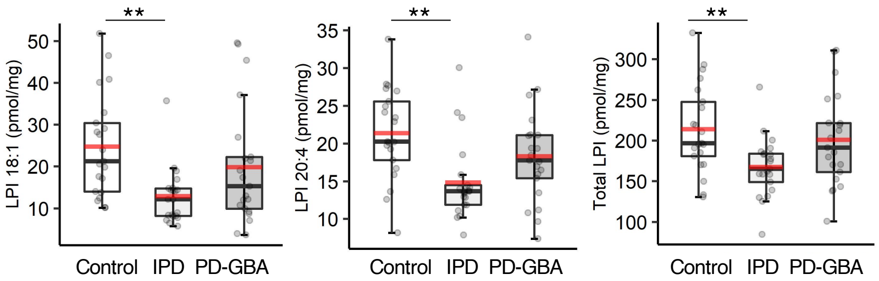
**
**
**
LPI 18:1 (pmol/mg)
50
40
30
20
10
LPI 20:4 (pmol/mg)
35
30
25
20
15
10
Total LPI (pmol/mg)
300
250
200
150
100
Control
IPD
PD-GBA
Control
IPD
PD-GBA
Control
IPD
PD-GBA

**Supplementary Figure 3. GD1a in IPD and PD-GBA brain.** Boxplots of concentrations of individual GD1a species (pmol/mg protein) along with the sum of all species (Total). The box represents lower quartile, median and upper quartile (*black*). The whiskers represent the minimum and maximum values, up to 1.5 times the interquartile range from the bottom or the top of the box to the furthest data point within that distance, thus excluding outliers. The mean is shown in *red*. *$p \leq 0.05$; **$p \leq 0.01$; ***$p \leq 0.001$.

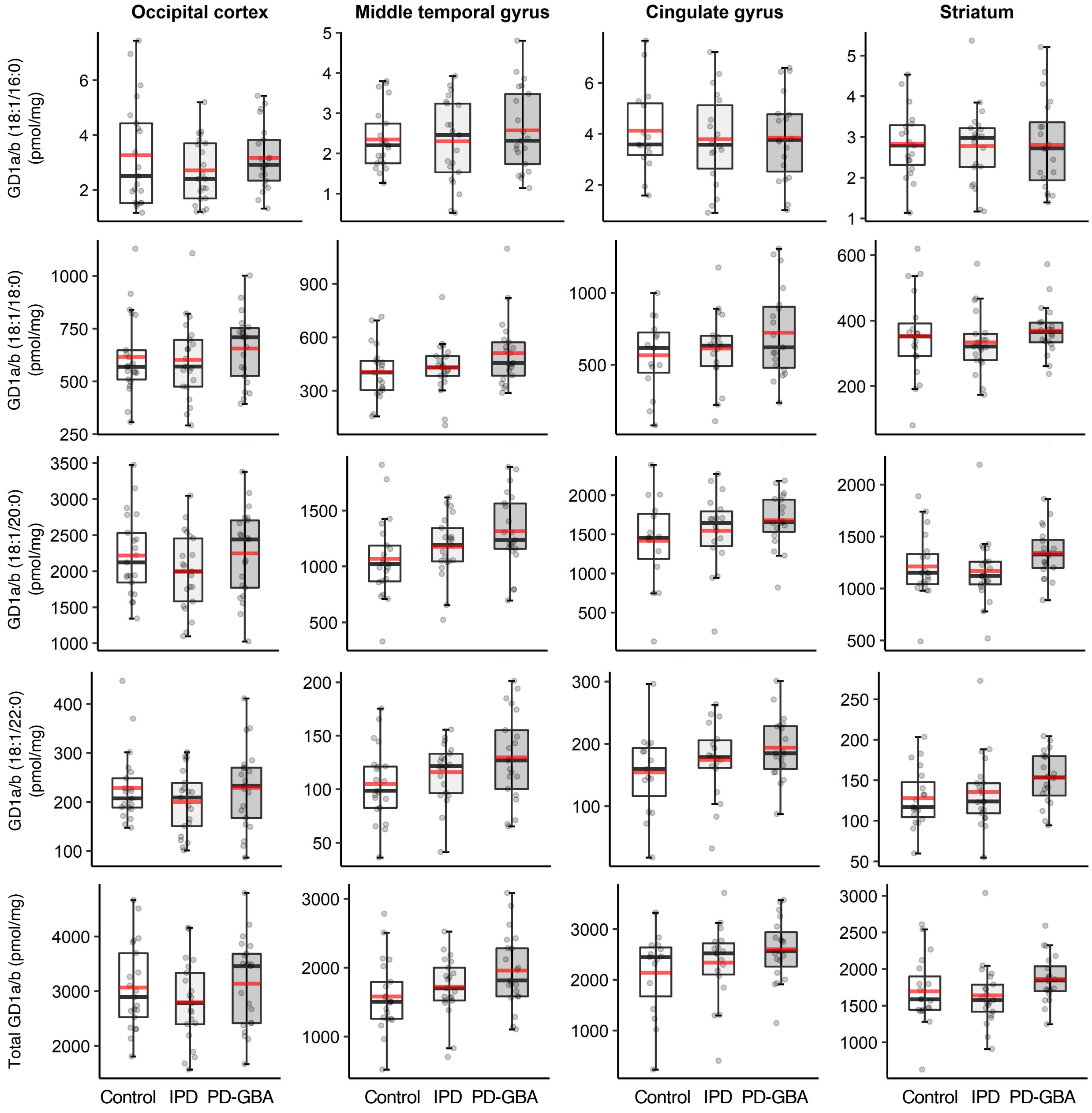

**Supplementary Figure 4. GD2 in IPD and PD-GBA brain.** Boxplots of concentrations of individual GD2 species (pmol/mg protein) along with the sum of all species (Total). The box represents lower quartile, median and upper quartile (*black*). The whiskers represent the minimum and maximum values, up to 1.5 times the interquartile range from the bottom or the top of the box to the furthest data point within that distance, thus excluding outliers. The mean is shown in *red*. *$p \leq 0.05$; **$p \leq 0.01$; ***$p \leq 0.001$.

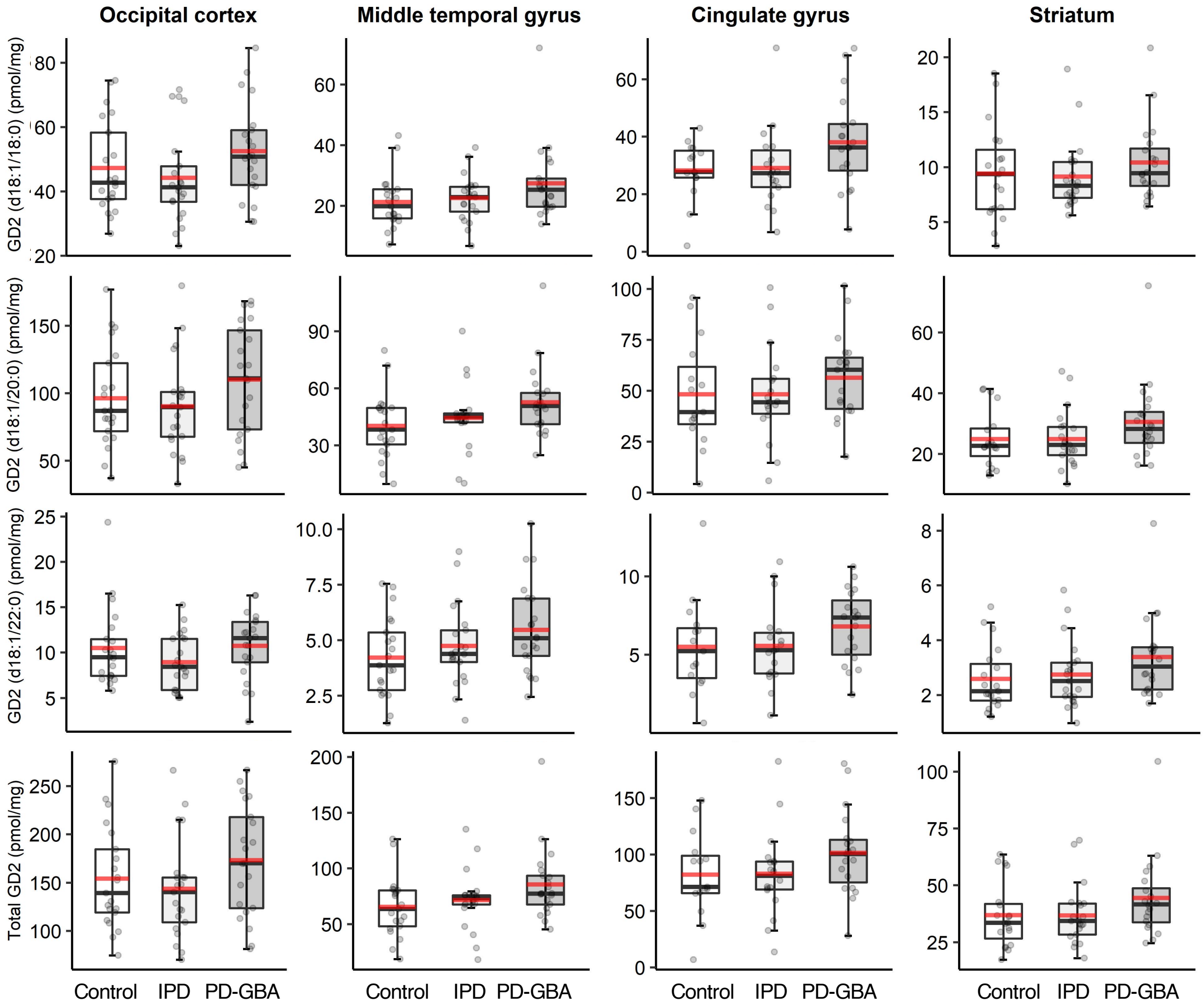

**Supplementary Figure 5. GD3 in IPD and PD-GBA brain.** Boxplots of concentrations of individual GD3 species (pmol/mg protein) along with the sum of all species (Total). The box represents lower quartile, median and upper quartile (*black*). The whiskers represent the minimum and maximum values, up to 1.5 times the interquartile range from the bottom or the top of the box to the furthest data point within that distance, thus excluding outliers. The mean is shown in *red*. *$p \leq 0.05$; **$p \leq 0.01$; ***$p \leq 0.001$.

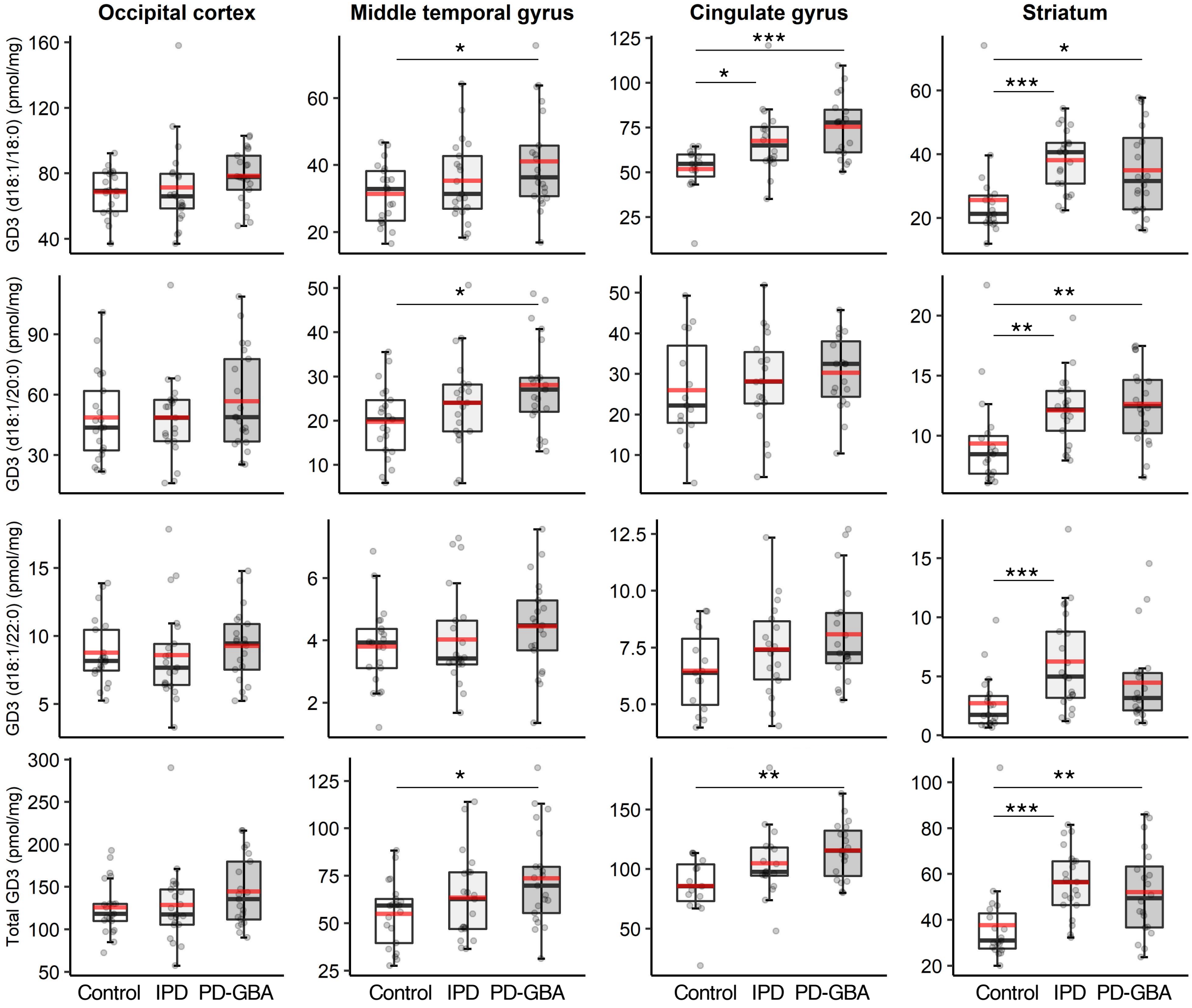

**Supplementary Figure 6. GM1 in IPD and PD-GBA brain.** Boxplots of concentrations of individual GM1 species (pmol/mg protein) along with the sum of all species (Total). The box represents lower quartile, median and upper quartile (*black*). The whiskers represent the minimum and maximum values, up to 1.5 times the interquartile range from the bottom or the top of the box to the furthest data point within that distance, thus excluding outliers. The mean is shown in *red*. **p* ≤ 0.05; ***p* ≤ 0.01; ****p* ≤ 0.001.

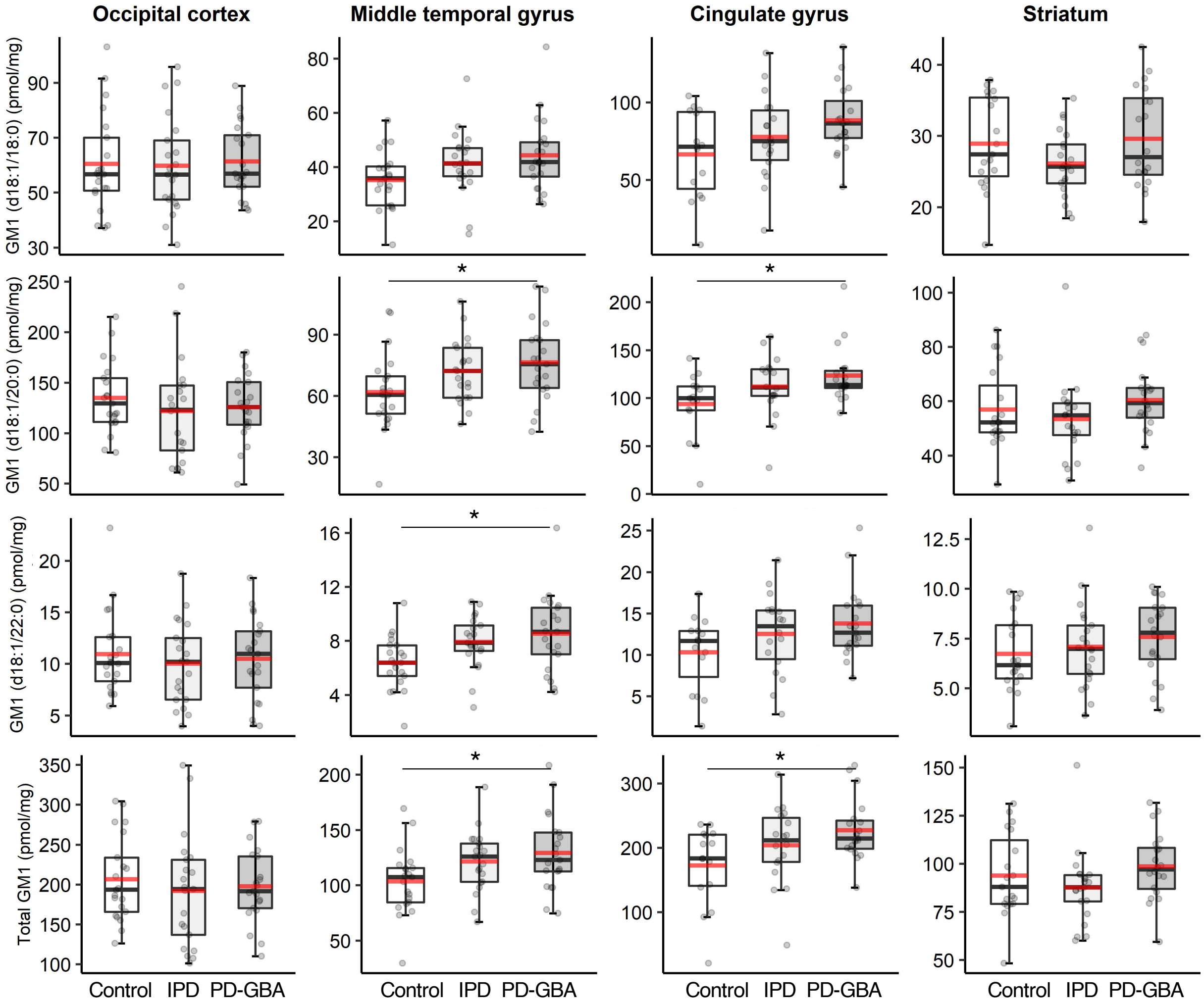

**Supplementary Figure 7. GM2 in IPD and PD-GBA brain.** Boxplots of concentrations of individual GM2 species (pmol/mg protein) along with the sum of all species (Total). The box represents lower quartile, median and upper quartile (*black*). The whiskers represent the minimum and maximum values, up to 1.5 times the interquartile range from the bottom or the top of the box to the furthest data point within that distance, thus excluding outliers. The mean is shown in *red*. *$p \leq 0.05$; **$p \leq 0.01$; ***$p \leq 0.001$.

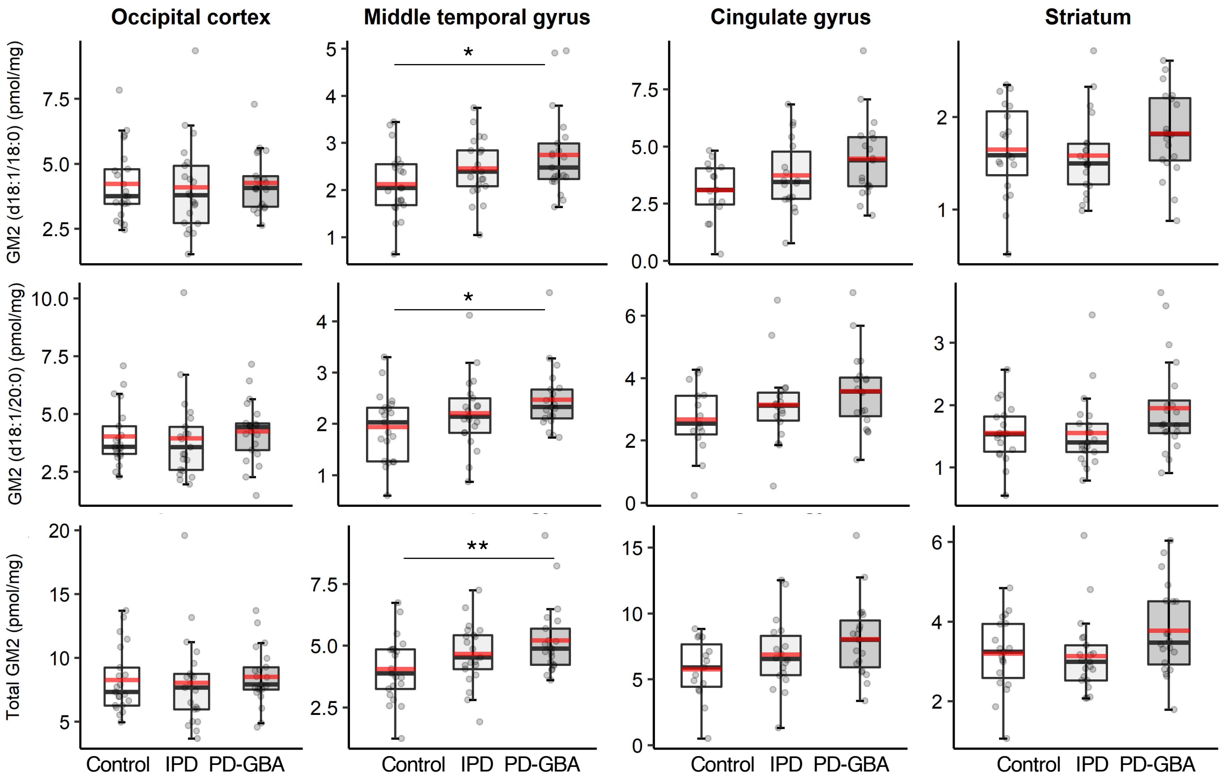

**Supplementary Figure 8. GM3 in IPD and PD-GBA brain.** Boxplots of concentrations of individual GM3 species (pmol/mg protein) along with the sum of all species (Total). The box represents lower quartile, median and upper quartile (*black*). The whiskers represent the minimum and maximum values, up to 1.5 times the interquartile range from the bottom or the top of the box to the furthest data point within that distance, thus excluding outliers. The mean is shown in *red*. *$p \leq 0.05$; **$p \leq 0.01$; ***$p \leq 0.001$.

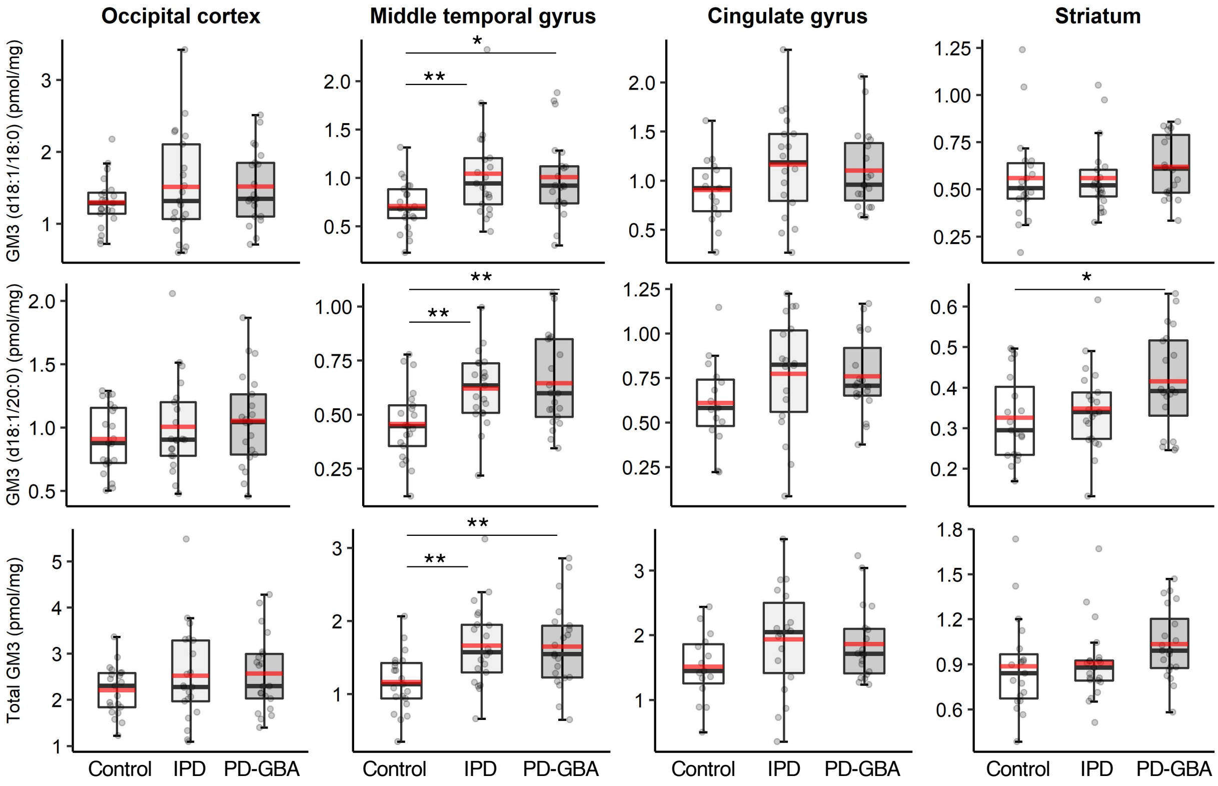

Supplement: Supplementary file 1 — Supplemental Material [file 41531_2022_363_MOESM1_ESM.pdf]
